# Chemical and Pharmacological Aspects of Caffeic Acid and Its Activity in Hepatocarcinoma

**DOI:** 10.3389/fonc.2019.00541

**Published:** 2019-06-21

**Authors:** Kaio Murilo Monteiro Espíndola, Roseane Guimarães Ferreira, Luis Eduardo Mosquera Narvaez, Amanda Caroline Rocha Silva Rosario, Agnes Hanna Machado da Silva, Ana Gabrielle Bispo Silva, Ana Paula Oliveira Vieira, Marta Chagas Monteiro

**Affiliations:** ^1^Laboratory of In Vitro Tests, Immunology and Microbiology-LABEIM, Exact and Natural Sciences Institute, Federal University of Pará/UFPA, Belém, Brazil; ^2^Laboratory of In Vitro Tests, Immunology and Microbiology-LABEIM, Biological Sciences Institute, Federal University of Pará/UFPA, Belém, Brazil; ^3^Laboratory of In Vitro Tests, Immunology and Microbiology-LABEIM, Health Science Institute, Federal University of Pará/UFPA, Belém, Brazil

**Keywords:** caffeic acid, anticarcinogenic activity, hepatocarcinoma, antioxidant activity, catechol group

## Abstract

Caffeic acid (CA) is a phenolic compound synthesized by all plant species and is present in foods such as coffee, wine, tea, and popular medicines such as propolis. This phenolic acid and its derivatives have antioxidant, anti-inflammatory and anticarcinogenic activity. *In vitro* and *in vivo* studies have demonstrated the anticarcinogenic activity of this compound against an important type of cancer, hepatocarcinoma (HCC), considered to be of high incidence, highly aggressive and causing considerable mortality across the world. The anticancer properties of CA are associated with its antioxidant and pro-oxidant capacity, attributed to its chemical structure that has free phenolic hydroxyls, the number and position of OH in the catechol group and the double bond in the carbonic chain. Pharmacokinetic studies indicate that this compound is hydrolyzed by the microflora of colonies and metabolized mainly in the intestinal mucosa through phase II enzymes, submitted to conjugation and methylation processes, forming sulphated, glucuronic and/or methylated conjugates by the action of sulfotransferases, UDP-glucotransferases, and o-methyltransferases, respectively. The transmembrane flux of CA in intestinal cells occurs through active transport mediated by monocarboxylic acid carriers. CA can act by preventing the production of ROS (reactive oxygen species), inducing DNA oxidation of cancer cells, as well as reducing tumor cell angiogenesis, blocking STATS (transcription factor and signal translation 3) and suppression of MMP2 and MMP-9 (collagen IV metalloproteases). Thus, this review provides an overview of the chemical and pharmacological parameters of CA and its derivatives, demonstrating its mechanism of action and pharmacokinetic aspects, as well as a critical analysis of its action in the fight against hepatocarcinoma.

## Introduction

Caffeic acid (CA) is a polyphenol produced through the secondary metabolism of vegetables, (1–4) including olives, coffee beans, fruits, potatoes, carrots and propolis, and constitutes the main hydroxycinnamic acid found in the diet of humans (1, 3–5). This phenolic compound is found in the simple form (monomers) as organic acid esters, sugar esters, amides and glycosides, or in more complex forms such as dimers, trimers and flavonoid derivatives, or they may also be bound to proteins and other polymers in the cell wall of the vegetable (1, 3, 6). CA participates in the defense mechanism of plants against predators, pests and infections, as it has an inhibitory effect on the growth of insects, fungi and bacteria (5) and also promote the protection of plant leaves against ultraviolet radiation B (UV-B) (5, 7).

*In vitro* and *in vivo* experiments have been performed, proving innumerable physiological effects of CA and its derivatives, such as antibacterial activity (1, 4), antiviral activity (2, 5, 8, 9), antioxidant activity (2, 4, 5, 8, 9), anti-inflammatory activity (2, 4, 5, 8, 9), anti-atherosclerotic activity (1, 4), immunostimulatory activity (1, 10), antidiabetic activity (5, 9), cardioprotective activity (5, 11), antiproliferative activity (1, 12, 13), hepatoprotective activity (14, 15), anticancer activity (2, 4, 5, 8, 9), and anti-hepatocellular carcinoma activity (16–18). Among these properties, anti-hepatocarcinoma activity is highlighted, because hepatocarcinoma (HCC) is one of the main causes of cancer mortality in the world (19). Therefore, further studies on the chemical and pharmacological aspects of CA are necessary to contribute in the future to the development of a new drug and consequently the expansion of therapeutic possibilities (20). Thus, this review provides an overview of the chemical and pharmacological parameters of CA and its derivatives, reporting its main mechanisms of action and pharmacokinetic aspects, as well as to critically analyse its performance in the fight against HCC.

## Chemical Aspects of Caffeic Acid

**Figure 1:**
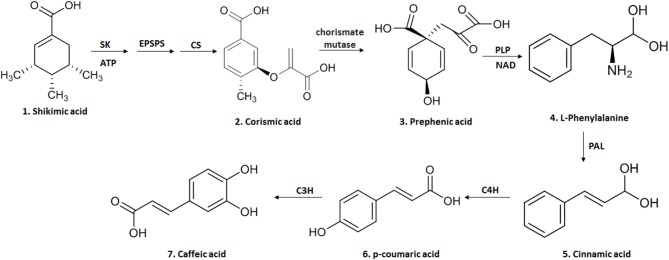
The biosynthesis of CA begins in the endogenous shikimate pathway through three enzymatic reactions mediated by shikimate kinase (KS), 5-enolpyruvyl-chiquimate-3-phosphate synthase (EPSPS) and chorismate synthase (CS), leading to chorismic acid and then converted into prephenic acid by chorismate mutase. Prephenic acid is a precursor of l-phenylalanine and formed by pyridoxal phosphate (PLP) and nicotinamide adenine (NAD). The deamination of L-phenylalanine by the enzyme phenylalanine ammonia lyase (PAL) forms cinnamic acid; this is then converted to p-coumaric acid by cinnamate-4-hydroxylase (C4H) and finally to caffeic acid through the enzyme 4-coumarate 3-hydroxylase (C3H). This figure was made with ChemDraw (http://www.perkinelmer.com/category/chemdraw).

AC (3,4-dihydroxycinnamic acid) is a hydroxycinnamic acid, belonging to the phenolic acid family, which has a phenylpropanoid (C6-C3) structure with a 3,4-dihydroxylated aromatic ring attached to a carboxylic acid through a transethylene wire (3, 21). The biosynthesis of this compound in plants occurs through the endogenous shikimate pathway that is responsible for the production of aromatic amino acids from glucose (3, 9). The reaction starts with shikimic acid and undergoes three enzymatic reactions: the first is a phosphorylation mediated by the enzyme shikimato-kinase, followed by the conjugation of a molecule of phosphoenolpyruvate, mediated by 5-enolpyruvylshikimate-3-phosphate (EPSP) synthase and finally by the enzyme chorismate synthetase, reaching one of the most important intermediary metabolites of this pathway, chorismic acid (3, 9). This is transformed into prephenic acid through the enzyme chorismate mutase (a precursor of L-phenylalanine). L-phenylalanine formation is mediated by pyridoxal phosphate (PLP) as a coenzyme in the deamination process and by nicotinamide adenine dinucleotide (NAD) as an electron exchanger (3, 9). The deamination of L-phenylalanine by the enzyme phenylalanine ammonia lyase (PAL), forms cinnamic acid. It is then converted to p-coumaric acid by the cinnamate-4-hydroxylase (C4H) and finally to caffeic acid through the enzyme 4-coumarate 3-hydroxylase (C3H) (9) (Figure 1).

**Figure 2:**
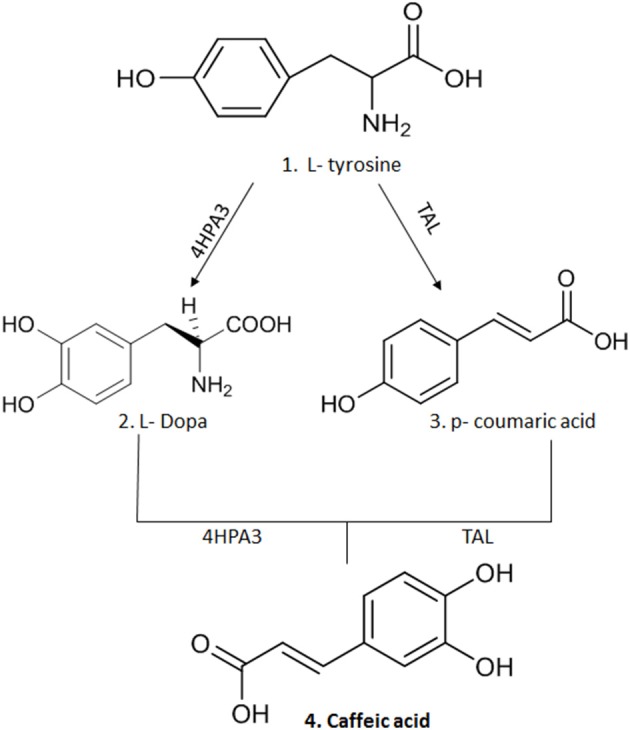
The synthesis of CA after genetic modification in strains of *E.coli* that allowed the production of two enzymes: 3-hydroxylase hydroxyphenylacetate (4HPA3H) and tyrosine ammonia lyase (TAL) that act on L-tyrosine producing p-coumaric acid and L-dopa, respectively. A new action of these enzymes on the two intermediate molecules leads to production of CA. This figure was made with ChemDraw (http://www.perkinelmer.com/category/chemdraw).

CA is obtained from plants by solvent extraction (methanol and ethyl acetate) at high temperatures; however, its yield is very low, requiring large quantities of botanical material to obtain a significant yield (8, 9). An alternative to obtain this compound in greater quantity is organic synthesis, where chemical processes (chemical reactions) are used that allow the formation of complex organic compounds (22). However, the concern with the generation of residues in the production of organic compounds has brought about the possibility of microbial synthesis of secondary metabolites such as CA (2, 8). Thus, genetic modifications made in microorganisms, such as Escherichia coli strains, have allowed the production of two enzymes: 3-hydroxylase hydroxyphenylacetate (4HPA3H) and tyrosine ammonia lyase (TAL) that acts on L-tyrosine producing p-coumaric acid and L-dopa, respectively. A new action of these enzymes on the two intermediate molecules leads to the generation of CA (2, 8, 9) (Figure 2).

## Pharmacokinetics of Caffeic Acid

CA is a very abundant phenolic acid, found in both free and esterified forms, representing about 75 to 100% of the total content of hydroxycinnamic acid in fruits (23). However, CA is found in foods on its esterified form, making it difficult to be absorbed by the body (23–25). To be absorbed, the compound needs to be hydrolysed by colonic microflora in the intestine, because human tissues (intestinal mucosa, liver, stomach) and biological fluids (plasma, gastric juice, duodenal fluid) do not have enzymes, called esterases, capable of hydrolysing the chlorogenic acid to release CA (23–25). Thus, the pharmacokinetic process begins with the ingestion of CA in the bound form (esterified), arriving in the stomach, after which a small part of this compound is absorbed (26). In the colon the microbial esterases cleave the ester portion of the CA and this acid, in its free form, is then absorbed by the intestinal mucosa (most 95%) (26). The transmembrane flow of CA into intestinal cells occurs through active transport mediated by monocarboxylic acid transporters (MCT) (26). The maximum plasma concentration of this compound was observed only 1 h after ingestion of foods such as coffee and then plasma concentration rapidly decreased, requiring repeated doses every 2 h to maintain high concentrations (23–25). Immediately after absorption, CA is subjected to three main processes of enzymatic conjugation (known as detoxification): methylation, sulphation, and glucuronidation, through the action of sulfotransferase enzymes, UDP-glucotransferases and catechol-o-methyltransferases, respectively. This makes the compound more hydrophilic, thus reducing its toxicity and facilitating its elimination (26). The excretion of CA (5.9–27%) occurs primarily through urine (23).

## Anticarcinogenic Properties of Caffeic Acid

The anticarcinogenic properties of CA have attracted the attention of the scientific community (27–30). Studies have shown that the consumption of CA-rich foods leads to a protective effect against carcinogenesis by preventing the formation of nitro compounds (nitrosamines and nitrosamides) that are the main inducers of this pathology (29, 31). The anti-carcinogenic action of CA is mainly associated with its antioxidant (21, 30, 32) and pro-oxidant capacities (33–35), and this is attributed to its chemical structure. Firstly, the presence of free phenolic hydroxyls (ortho-dihydroxyl) makes it possible to reduce the enthalpy of OH-bond dissociation and increase the transfer rate of H atoms for peroxyl radicals, as well as their number and position on the phenyl ring (catechol group). Also, the presence of a double bond in the carbon chain (the unsaturated side chain 2,3 double bond) increases the stability of the phenolic radical (21, 30, 31, 36). These chemical factors associated with the CA molecule enable the elimination of free radicals, preventing the production of ROS (reactive oxygen species) as well as the induction of DNA oxidation of cancer cells present in various types of cancer, such as HCC (3, 30).

## Hepatocarcinoma (HCC)

Hepatocarcinoma or Hepatocellular Carcinoma (HCC) is a dominant form of liver cancer, characterized by being a malignant primary solid tumor, which differs from hepatocytes (19, 37, 38), and is considered the third most common cause of cancer death, second only to lung and stomach cancer (19). HCC is a very aggressive disease, with approximately 782,000 new cases per year, a high mortality (600,000 deaths/year) (19, 39, 40) and a short survival time (on average 11 months). It is thought this is because only a small proportion of the patients are diagnosed at the initial stage of the disease (19, 41). HCC has several risk factors, such as exposure to hepatitis B virus (HBV) and C (HCV), aflatoxin B1 (AFB1), presence of cirrhosis, alcohol consumption, diabetes mellitus and obesity (19, 38, 40, 42). These factors vary in their frequency according to geographic location. Among the cited causes, infection with the HBV and HCV virus is considered the main risk factor, and it is worth noting that 80% of cases occur in Southeast Asia and Africa while the Western world accounts for only 20% of cases (19, 42). The high incidence of HCC in the Asian and African continent is associated with the HBV and HCV virus, as well as exposure to food contaminated by AFB1, especially in rural areas where there is no strict control on mycotoxin contamination (42–44). However, synergism among risk factors is what makes these regions highly prevalent to HCC (42, 45).

The mechanism of HCC pathophysiology begins with an inflammatory process mediated by Kupffer cells in the liver or macrophages (46, 47), both of which have immunostimulatory activity, secreting pro-inflammatory cytokines (interleukin 6-IL6 and TNF-α-tumor necrosis factor) and immunosuppressive cytokines (interleukin 10-IL-10) (46, 47). The accumulation of these immunostimulating agents around the focus of inflammation in the liver induces a mitochondrial imbalance in hepatocytes (increased oxygen consumption and increased production of superoxide anions, hydroxyl radicals and nitric oxide), leading to the production of high levels of reactive oxygen species (ROS) (48–50). Hepatic mitochondria control the balance between survival and cell death through the regulation of membrane permeability, activating the intrinsic pathway of apoptosis (release of cytochrome c protein, formation of apoptosomes and activation of caspase 9) (49, 51). Increased ROS production causes oxidative damage to mitochondrial proteins (impairing ATP synthesis) and alters the induction of mitochondrial transition permeability pore production by making the inner membrane permeable to small molecules that can cause ischaemia/reperfusion injury, as well as DNA damage by activating the apoptotic intrinsic mechanism (49, 51). This process induces recurrent cycles of cell injury, repair and regeneration in hepatocytes leading to the formation of nodular dysplasia (aberrant hepatocytes), which are precursor lesions of HCC (40, 42, 52). It also leads to genetic alterations (gene rearrangements, somatic mutations, changes in copy number, changes in cell signaling pathways) and epigenetic changes (DNA methylation and histone modification), which are directly associated with tumor progression and HCC (35, 40).

Patients with this pathology are usually asymptomatic; however, it is associated with the appearance of abdominal pain, fever, hyperkalaemia, hypoglycaemia, nausea, weight loss, increased ascites, spontaneous bacterial peritonitis, varicose veins, bleeding, jaundice, and hepatic encephalopathy (53, 54). The aggravation of liver function is due to functional liver replacement or portal vein invasion (53, 54). Treatment for HCC is limited due to the complex etiology of the disease and, mainly, the toxicity of the drugs to normal cells, making it necessary to develop new drugs for the therapy of this cancer (20, 38). Thus, the investigation of mechanisms of action and characterization of the hepatoprotective and anticarcinogenic action of CA can help support the future development of new therapies to combat this disease (20).

## Caffeic Acid Action Mechanism in HCC

### Antioxidant Activity Prevents ROS Production

**Figure 3:**
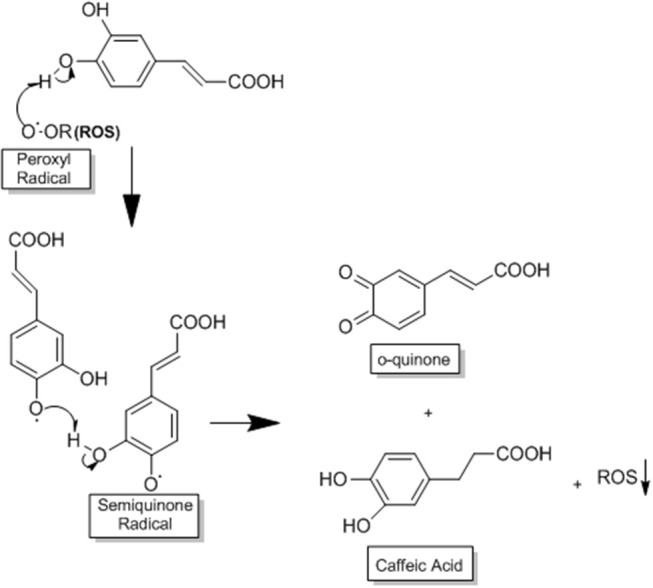
This process occurs when CA donates electrons or hydrogen to free radicals, converting them into thermodynamically stable products. These products present greater stability due to the electron delocalised in the aromatic ring of CA (resonance effect). The o-dihydroxylated structure is where the o-quinone group is produced after the donation of an electron. The double side binding of o-quinone conjugated to the catechol group causes a delocalisation of electrons, increasing the stability of the o-quinone radical and the antioxidant activity of CA, adapted from: Damasceno et al. (29). This figure was made with ChemDraw (http://www.perkinelmer.com/category/chemdraw).

One of the mechanisms by which CA acts in HCC is through its potent antioxidant capacity that prevents the production of ROS, reducing the oxidative stress that is very common in this disease (3, 30). CA acts as a primary and secondary antioxidant (mixed antioxidant). As a primary antioxidant it acts by interrupting the formation of free radicals by inhibiting chain reactions with another molecule (29, 55). This process occurs when CA donates electrons or hydrogen to free radicals, converting them into thermodynamically stable products. These products present greater stability due to the electron delocalised in the aromatic ring of the CA (resonance effect) (29). In contrast, as a secondary antioxidant (prooxidant), it acts as a chelating agent. It forms complexes with metals (mainly iron and copper), inhibiting the decomposition of peroxides, reducing the formation of free radicals and their attack on lipids, amino acids of proteins, double bonding of polyunsaturated fatty acids and bases of DNA, thus avoiding the formation of lesions and loss of cellular integrity (29, 55). CA has a great potential for reducing metals due to its structural chemical characteristics; the compound is susceptible to auto-oxidation and also the oxidation caused by other biological agents (29, 56). The molecular structure of CA, containing a catechol group with an α,β-unsaturated carboxylic acid chain, is responsible for its interaction with various types of oxidizing radicals (29, 56). The o-dihydroxylated structure is where the o-quinone group is produced after the donation of an electron. The double side binding of o-quinone conjugated to the catechol group causes a delocalisation of electrons, increasing the stability of the o-quinone radical and the antioxidant activity of CA (29, 56) (Figure 3).

### Pro-oxidant Activity Accelerates Lipid Peroxidation and DNA Damage

**Figure 4:**
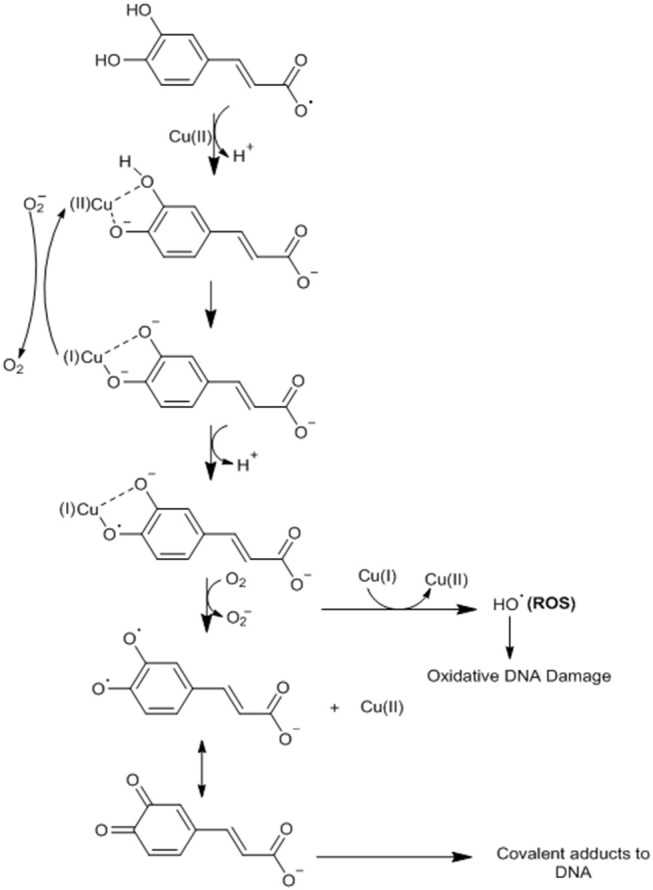
CA undergoes deprotonation in relation to Cu, generating an oxygen center with high electronic density. This complex undergoes intramolecular electron transfer (oxygen) forming the semiquinone radical anion with Cu (I). CA dissociates (deprotonation) to form a phenoxide, where the Cu (I) ion will be bound as a bidentate linker. At this point, oxygen (O_2_) can react with Cu (I) to form hydrogen peroxide (H_2_O_2_), which is converted by a Fenton reaction (reactions with H_2_O_2_ catalyzed by Fe ions) to the hydroxyl radical (H_2_OH) or the phenoxide bound to bidentate Cu (I) may undergo a new intramolecular transfer giving the orthohydroxyphenoxyl radical, which must dissociate and form ortho-semiquinone anions. This anion reacts with Cu (I), generating the final ortho-quinone product that forms a covalent adduct with the DNA of cancer cells. Adapted from: Damasceno et al. (29). This figure was made with ChemDraw (http://www.perkinelmer.com/category/chemdraw).

An antioxidant agent such as CA can become a pro-oxidant through its ability to chelate metals such as copper (Cu) and thus induce lipid peroxidation and causing damage to the DNA of cancer cells by oxidation or formation of covalent adducts with DNA (29, 57). CA possesses the ability to cap the endogenous Cu (II) ions of human peripheral lymphocytes to form the CA-Cu (II) complex (57). CA undergoes deprotonation in relation to Cu, generating an oxygen center with high electronic density (29, 57). This complex undergoes intramolecular electron transfer (oxygen) forming the semiquinone radical anion with Cu (I) (29, 57). CA dissociates (deprotonation) to form a phenoxide, where the Cu (I) ion will be bound as a bidentate linker (29, 57). At this point, the oxygen (O_2_) can react with Cu (I) to form the hydrogen peroxide (H_2_O_2_), which is converted by a Fenton reaction (reactions with H_2_O_2_ catalyzed by Fe ions) to the hydroxyl radical (^−^OH) (29, 57). Alternatively, the phenoxide bound to bidentate Cu (I) may undergo a new intramolecular transfer giving the orthohydroxyphenoxyl radical, which must dissociate and form ortho-semiquinone anionsz (29, 57). This anion reacts with Cu (I), generating the final ortho-quinone product that forms a covalent adduct with the DNA of cancer cells (29, 57) (Figure 4).

### Vascularisation Induced by VEGF

**Figure 5:**
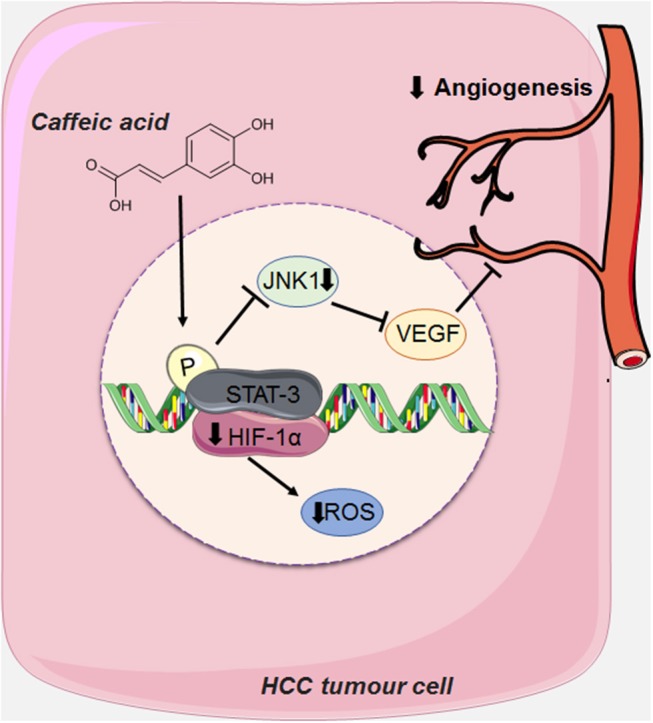
CA acts on the angioneogenesis of HCC tumor cells by reducing the phosphorylation of JNK-1, thus decreasing the activation of HIF-1α (Hypoxia Inducible Factor 1), causing the reduction of the intense vascularisation induced by VEGF (endothelial growth factor vascular). This figure used elements from Servier Medical Art (www.servier.com).

CA can also act on angioneogenesis of HCC tumor cells by reducing the phosphorylation of JNK-1 (c-Jun N-terminal kinases, a member of the MAPKs family), via decreasing the activation of HIF-1α (Hypoxia Inducible Factor 1). This leads to the reduction of vascularisation induced by VEGF (vascular endothelial growth factor), and consequently suppressing tumor growth (18) (Figure 5).

HCC is a highly vascularised tumor, whose main characteristic is angioneogenesis (the origin of new blood vessels), and its main source of blood supply is the hepatic artery (18, 40, 58). Although this tumor is rich in vascularisation, hypoxia (low levels of oxygen) is very common due to the rapid proliferation of tumor cells and consequently the formation of large solid tumor masses, obstructing and compressing the blood vessels around it (18, 40, 58). Tumor cells seek to adapt to hypoxia by activating a transcription factor called HIF-1 (Hypoxia Inducible Factor 1) via the JNK1 signaling pathway (c-Jun N-terminal kinases, a member of the MAPKs family), which in turn, activates several pro-angiogenic factors, such as VEGF (vascular endothelial growth factor). When overexpressed VEGF leads to extravasation of blood from tumor blood vessels, causing hepatic bleeding (18, 59). This growth factor is also one of the main factors responsible for the formation of new blood vessels that support the oxygen supply of tumor cells, an important factor for tumor survival (18, 40, 58, 59).

### Suppression of MMP-2 and MMP-9 Expression

**Figure 6:**
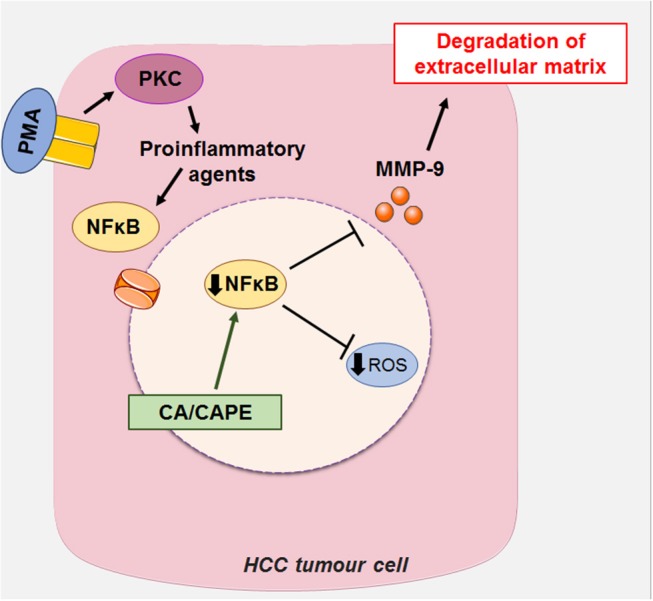
CA, along with its caffeic acid phenyl ester (CAPE), can act on HCC by suppressing the expression of MMP-2 and MMP-9 (metalloproteinases 2 and 9, respectively), which in turn block the activation of NFκB (Nuclear factor kappa-β) induced by PMA (activating protein 1) in liver cancer cells, decreasing tumor growth and invasiveness. This figure used elements from Servier Medical Art (www.servier.com).

Another mechanism of action proposed for CA is the suppression of the expression of MMP-2 and MMP-9 (metalloproteases 2 and 9, respectively) in HCC. MMP-2 and MMP-9 are matrix metalloproteases expressed in tumor cells, which degrade the extracellular matrix (ECM) type IV collagen of the basement membrane during cancer invasion and metastasis (16, 60, 61). PMA is a structural analog of diacylglycerol (a cellular mediator) capable of activating PKC (kinase C protein), which, once activated, promotes the induction of proinflammatory cytokines such as IL-6 (interleukin 6) and TNF-α (tumor necrosis factor α) (62). These pro-inflammatory agents promote the activation of growth factors, such as NFκB (nuclear factor kappa-B) through the c-Src (subfamily kinases)/ERK (Extracellular Signal-Regulated Protein Kinase)/NIK (NSP-interacting kinases)/IKK (I kappa B kinase) (14, 31, 63). NFkB generates increased expression of MMP-2 and MMP-9 leading to invasion and metastasis of hepatic cells via degradation of ECM (16, 60, 61). The suppressive effect of CA on MMP-2 and MMP-9 is associated with blockade of the activation of NFkB, as reported in liver cancer cells stimulated by PMA (activating protein 1), leading to a decrease in tumor growth and invasiveness (16, 60) (Figure 6).

## *In vitro* and *In vivo* Studies of Caffeic Acid in Hepatocarcinoma

### In vitro

Dilshara et al. showed that CAPE (Caffeic acid phenethyl ester, 50 μM) significantly increased apoptosis (cell death) mediated by TRAIL (tumor necrosis factor-related apoptosis ligand inducer, 50 ng/ml) by positive regulation of DR5 (death receptor 5) mediated by CHOP (C / EBP family transcription factor) in Hep3B HCC cells. TRAIL is a ligand with anticancer properties capable of inducing apoptosis in cancer cells (64, 65). This action occurs through its binding to DR5 (extrinsic pathway), which in turn interacts with Fas (membrane protein) by recruiting caspases 8 and caspase 3 (cysteine proteases) and promoting apoptosis (65, 66). In this study, CAPE, a derivative of CA present in the bee propolis extract, potentiated TRAIL-mediated cell death, stimulating the expression of the CHOP protein, responsible for the DR5 positive regulation (65).

In another experiment developed by Kim et al., the CAPE (30 μg/ml) potentiated TRAIL-induced apoptosis (30 ng/ml) through the positive regulation of DR5 via p38 (mitogen-activated protein kinases) and suppression of JNK (c-Jun N-terminal kinases) in SK-Hep1 cells of HCC (14, 63).

The combination of CAPE and TRAIL was able to generate cell death both via intrinsic pathway (via mitochondria) and via the extrinsic pathway (via death receptors) (14). In the intrinsic CAPE and TRAIL pathway, mitochondrial membrane depolarisation stimuli were increased, resulting in the release of cytochrome c (internal membrane protein from mitochondria) and formation of the apoptosome (protein complex), and also resulting in the activation of apoptosis-inducing caspase 9 (14, 67). On the other hand, through the extrinsic pathway, CAPE and TRAIL promoted the activation of p38 by increasing the expression of apoptosis-inducing DR5, as well as inhibiting the phosphorylation of JNK that contributes to TRAIL resistance and, consequently, decreased DR5 expression (14, 68).

Wilkins et al., in their experiments, observed that CA (1 mM) blocked cell proliferation in HCC cells extracted from marmots. The action of this compound is associated with its involvement in the loss of mitochondrial integrity (intrinsic pathway), resulting in cytochrome c release, apoptosome formation, caspase 9 activation and cell death (69).

Lee et al. showed that CAPE (12.5 μM) inhibited the invasion and expression of MMP-2 and MMP-9 (enzymes responsible for extracellular matrix degradation and development of metastasis) in HCC cells (SK-Hep1) blockade of NFkB (protein complex responsible for the regulation of MMP-2 and MMP-9) (16). In other studies, Guerriero et al., the CA (200 μg / ml) inhibited tumor invasion and regression in HCC cells (HepG2 and Huh7) by decreasing pro inflammatory cytokines such as TNF-α, IL-1β, and IL-8 and anti-inflammatory cytokines, such as IL-10 (70).

Li et al. showed that the conjugated CA and its precursor's cinnamic acid and p-coumaric acid to TPP cations (triphenylphosphonium) protected the hepatic mitochondria (key organelle in the control of apoptosis) against lipid peroxidation, also acting in the decrease of levels of hydrogen peroxide and the regulation of antioxidant enzymes. These same compounds were tested on HCC cells (HepG2), showing high toxicity against this cell line. Studies on CA and its effects on HCC are abundant in the literature, however, the same is not true for the action of CA and p-COA on HCC (71).

### In vivo

Zhang et al. investigated the action of CA (100 mg/kg) on structural changes caused by HCC in the rat microbiota, demonstrating that this compound reduces the changes in markers of liver injury during HCC, such as alanine, transaminase, aspartate aminotransferase, alkaline phosphatase, total bile acid and total cholesterol. The probable mechanism by which CA acts is related to inhibition of the growth of malefic bacteria (Rumincoccaceae UCG-004) (20, 72) and induction of the growth of microbiota-beneficial bacteria (Lachnospiraceae incertae sedis and Prevotella 9) during HCC (20, 73). CA has antioxidant properties, being able to eliminate oxygen radicals and facilitating the survival of beneficial bacteria that are anaerobic and grow very well without oxygen (20, 74). However, this same compound also has antimicrobial activity eliminating malefic bacteria of the microbiota, favoring the control of markers of liver damage (20, 75).

In studies conducted by Wilkins et al., the CA (1 mM) improved the efficacy of transarterial embolisation (TAE) in rats with HCC. TAE is a therapeutic procedure used in patients with HCC to promote ischemia due to occlusion of the arterial blood supply, resulting in the blockage of oxygen and nutrients for the tumor (69, 76). One of the main nutrients for HCC is lactate produced through glycolytic metabolism and is responsible for increasing the expression of vascular growth factors in vasculogenesis (69, 77). However, if lactate levels become excessively high, a cycle of negative feedback on glycolysis occurs, which stops tumor growth (69, 77, 78). CA used in association with TAE reduced tumor burden by 70–85%, compared to TAE only with embolic agents (69). This effect is possible due to the antitumor, anti-inflammatory, antioxidant and anticancer properties of this phenolic compound, which is capable of generating ROS and fragmenting DNA, causing cell death in cancer cells (69). CA may also activate the intrinsic pathway of apoptosis by altering the membrane potential of mitochondria (69, 79).

Chung et al. in experimental animal studies (rats) treated with CA and CAPE (5 mg/kg subcutaneous or 20 mg/kg oral), showed that these compounds promoted suppression of tumor growth in HCC cells (HepG2) as well as reduction of tumor invasion at a metastatic site in the liver. The authors suggest that both CA and CAPE inhibit and block the enzymatic activity of MMP-9 (causing invasion and cell metastasis), by inhibiting NFkB function (MMP-9 regulator) (60).

Macías-Pérez et al. demonstrated the chemoprotective effect of CAPE and its analogs LQM717 and LQM706 (20 mg / kg) on necrosis, lipid peroxidation, cell proliferation, p56 activation (protein tyrosine kinase, tumor suppressor) and alteration of hepatic tumors (HCC) in rats. The effects of these compounds on lipid peroxidation are attributed to the direct antioxidant activity (chelating properties and ROS decrease) of CAPE and the indirect antioxidant activity (inhibition of the metabolism or induction of the antioxidant system in the cells) of their analogs. In addition, the three compounds act by decreasing the activation of p53, NFkB activator (apoptosis-related transcription factor, proliferation and cell cycle) (80).

CA is a highly versatile compound with multiple biological activities impacting human health (antioxidant, hepatoprotection, antitumor, anti-inflammatory, antimicrobial) (1, 4, 5, 8, 9, 14, 15). This fact seems to favor its action in the HCC, since *in vitro* and *in vivo* studies already demonstrated its performance through several mechanisms of action in the fight against this disease, such as: ROS prevention (3, 30), prooxidant action (29, 57), angiogenesis (18), suppression of MMP-2 and MMP-9, (18, 60), justifying the differences in the results found.

Overall, *in vitro* and *in vivo* studies have shown that CA and its derivatives exerted their anti-hepatocarcinoma effect, dependent on various mechanisms such as apoptosis by induction of TRAIL pathway and caspase 9 activation, loss of integrity and depolarization of mitochondria, release of cytochrome c, and formation of the apoptosome. However, these mechanisms and the parameters evaluated varied greatly *in vitro* and *in vivo* studies, such as different concentrations and doses (30 μg/ml, 200 μg/ml 12.5 μM, 1 mM, 50 μM, 100 mg/kg, 20 mg/kg), different HCC cell lines (Hep3, SK-Hep1, HepG2), as well as different routes of administration used *in vivo* model (e.g., oral and subcutaneous). Although, *in vitro* and *in vivo* data have reaffirmed the promising role of this compound in HCC therapy, confirming the antitumor, anti-inflammatory, antioxidant, and anticancer properties of CA and its derivatives, but studies are needed to better elucidate the mechanisms and pathways involved in the performance of this compound. Accordingly, preclinical studies of pharmacokinetics and adverse reactions are required to determine the therapeutic index of CA prior to human testing in order to validate the benefits of using this compound in the control of HCC.

## Conclusion

HCC is one of the most lethal types of cancer in the world, so the focus on the research of new natural agents capable of containing proliferation and metastasis in this pathological process represents an important strategy for the prevention and treatment of this disease. This study sought to elucidate knowledge about the chemical and pharmacological aspects of CA as well as its effects on HCC. This compound demonstrates activity against HCC, preventing the exaggerated formation of ROS and assisting in the killing of tumor cells through DNA oxidation, as well as angioneogenesis by acting to reduce VEGF-induced vascularisation and suppression of MMP-2 and MMP expression−9. The anticancer activity of CA seems to be associated with its potent antioxidant and pro-oxidant activity attributed to its chemical structure with free phenolic hydroxyls, the number and position of OH in the catechol group and the double bond in the carbonic chain. Although these data demonstrate CA anti-hepatocarcinoma activity, further studies are needed to explain aspects of toxicity, interactions and efficacy that demonstrate safety in the use of this phenolic compound as a possible candidate in the treatment of HCC.

Therefore, we critically analyze and suggest that CA demonstrate an anti-hepatocarcinoma action; however, the HCC can change multiple pathways, and then, there is a need that the CA to act simultaneously for crosstalk between inhibitory and regulatory pathways to provide improvements in the development and progression of HCC. In addition, caution is required in therapies with natural products, as CA and resveratrol, due to the lack of studies have addressed the efficacy of CA in hepatocarcinoma in humans and animals model, making it difficult to obtain concrete evidence of the effect of this antioxidant. Thereby, these outcomes are limited by lack of standardization in the design and duration of treatment, and the lack of agreement on the effective and tolerated dose, intrinsic aspects of the patient, environmental factors, and characteristics of the compound studied are important for efficacy and therapeutic success. In addition, the major limitation currently facing is the lack of information from clinical studies that is weak and largely inconclusive. Thus, clinical trials are mainly conducted with volunteers, not reflecting the target population, the participants' age is quite broad, sample size is rarely calculated.as reported by our group in previous work (81, 82). Thus, we conclude that, to date, evidence based on *in vivo* studies in human and animals model are still insufficient, contradictory, and inconclusive, so we recommend that more studies and clinical trials should be performed to fully elucidate the beneficial effects of caffeic acid supplementation on hepatocarcinoma and in the body, as well as its toxic effects on human health. However, we emphasize that caffeic acid is promising in health promotion, not only for its antioxidant activities but also for its preventing the angioneogenesis and MMP-2 and MMP-9 expression. Thereby, further studies assessing other routes of administration or pharmaceutical formulations (i.e., nanoencapsulation) are required to improve the tissue-targeting concentration and allow caffeic acid to exert its biological activities in HCC.

## Author Contributions

All authors participated in the design of the study and drafted the manuscript. MM participated in the study coordination and helped to draft the manuscript. KE, RF, and LN have designed and prepared the manuscript figures. All authors read and approved the final manuscript.

### Conflict of Interest Statement

The authors declare that the research was conducted in the absence of any commercial or financial relationships that could be construed as a potential conflict of interest.
